# Improving protein content and quality by over-expressing artificially synthetic fusion proteins with high lysine and threonine constituent in rice plants

**DOI:** 10.1038/srep34427

**Published:** 2016-09-28

**Authors:** Shu-Ye Jiang, Ali Ma, Lifen Xie, Srinivasan Ramachandran

**Affiliations:** 1 Rice Functional Genomics Group, Temasek Life Sciences Laboratory, 1 Research Link, National University of Singapore 117604, Singapore

## Abstract

Rice grains are rich in starch but low in protein with very low level of both lysine and threonine. Thus, it is important to further improve protein quality and quantity, especially to increase lysine and threonine content in rice grains. We artificially synthesized two new genes by fusing endogenous rice genes with lysine (K)/threonine (T) motif (TKTKK) coding sequences. They were designated as *TKTKK1* and *TKTKK2* and their encoded proteins consist of 73.1% and 83.5% of lysine/threonine, respectively. These two genes were under the control of 35S promoter and were independently introduced into the rice genome to generate transgenic plants. Our data showed that overexpression of *TKTKK1* generated stable proteins with expected molecular weight and the transgenic rice seeds significantly increased lysine, threonine, total amino acids and crude protein content by 33.87%, 21.21%, 19.43% and 20.45%, respectively when compared with wild type control; significant improvement was also observed in transgenic rice seeds overexpressing *TKTKK2*. However, limited improvement in protein quality and quantity was observed in transgenic seeds carrying tandom array of these two new genes. Our data provide the basis and alternative strategy on further improving protein quality and quantity in other crops or vegetable plants by synthetic biology.

Rice grains are rich in carbohydrates (nearly 90%) but low in protein (around 10%). Its protein contains low level of lysine and threonine, which are two of nine essential amino acids for humanity and many animals as they are lack of enzymatic machinery for *de novo* synthesis of these amino acids^1^. Thus, increasing lysine and threonine content in rice grains will have a significant social and economic impact.

Various attempts to improve the content of protein and essential amino acids such as lysine and threonine have been carried out. At the early stage, natural or artificial mutants were employed to improve lysine content through traditional breeding. For example, the high lysine maize mutant *opaque2*^2^ was used as a parent line to develop the ‘quality protein maize’ with nearly doubled lysine content in seeds^3^. In rice, higher lysine plants (14%) were regenerated from calli subjected to inhibitory levels of lysine plus threonine^4^.

The second strategy to increase essential amino acids is by modifying biosynthetic and catabolic fluxes^5^,^6^,^7^,^8^. This strategy is successful for improving free lysine, threonine and methionine in some plants including tobacco^9^,^10^, canola^11^, soybean^11^ and Arabidopsis^12^,^13^. However, in maize, the accumulation was mainly observed in embryo but not in endosperm^14^,^15^,^16^. In rice and barley, the expression of the bacterial *DHPS* only slightly increased the content of free lysine^17^,^18^. To improve the lysine and threonine content in these crops, silencing of *LKR/SDH* by RNA interference (RNAi) was carried out in maize, which dramatically increased lysine content in seeds^14^,^19^. In rice, free lysine level could be increased up to ~12-fold in leaves and ~60-fold in seeds by over-expression *AK* and *DHPS* and silencing *LKR*/*SDH* by RNAi^8^.

The third strategy is to generate transgenic plants by over-expressing genes encoding the proteins with higher ratios of essential amino acids. At least 3 lysine-rich genes have been characterized for improving lysine content. Expression of these genes could increase the lysine accumulation up to 10–65% in maize^20^,^21^,^22^,^23^. In rice, endogenous genes *RLRH1* and *RLRH2* were characterized, which encode proteins with 14.7% and 20.6% of lysine in their amino acid composition, respectively^24^. They were used for improving lysine content in rice and transgenic plants showed up to 35% increase in its content^24^. Besides these naturally evolved lysine-rich encoding genes, artificially synthetic or modified genes were also used for lysine, methionine, or cysteine enrichment in potato^25^, tobacco^26^ or soybean^27^.

In addition to the above mentioned strategies, genetic manipulation of seed storage proteins (SSPs) has also been employed to improve protein quality and quantity in crops^28^,^29^,^30^. Generally, various strategies have been employed to improve protein content and quantity, especially to increase the essential amino acid content. However, reports showed that these strategies were accompanied by various disadvantages^7^,^24^. Possible healthy risk has been reported on the application of high free lysine transgenic maize^31^. In this study, we have developed the efficient strategy to further improve protein content and quantity, especially for lysine and threonine content in rice leaves and mature seeds.

## Results

### Designing of the synthetic genes by fusing lysine-threonine coding motifs to rice endogenous genes

**Figure 1:**
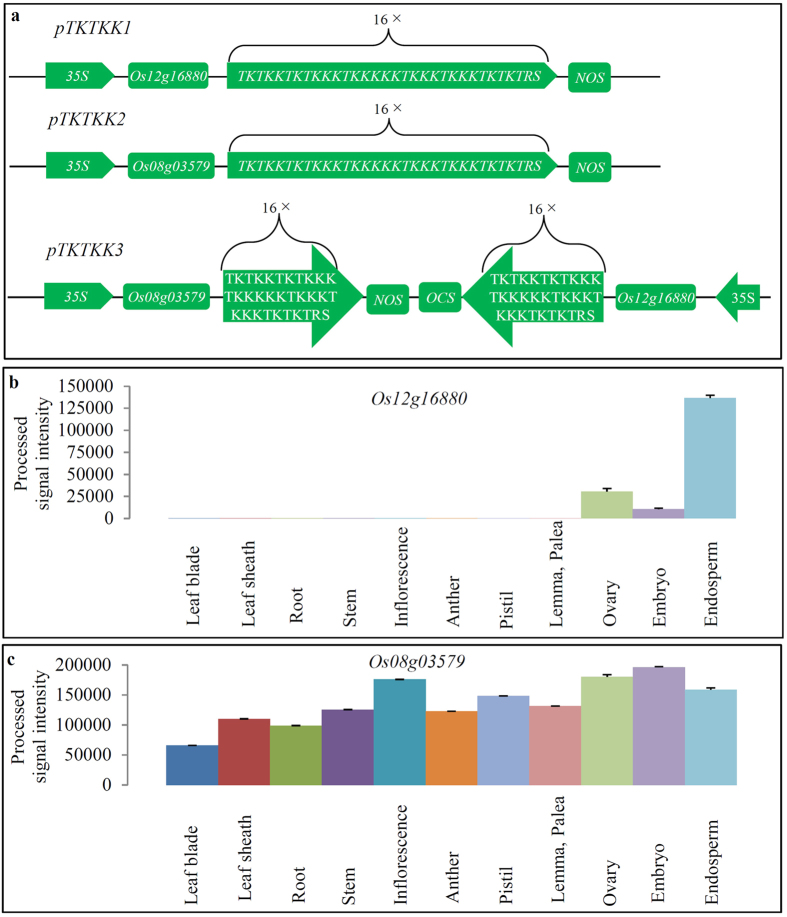
Construction of binary vectors by fusing endogenous rice genes with TKTKK coding motifs. **(a)** Construction of three binary vectors for improving lysine/threonine content. Top and middle panels showed the vectors *pTKTKK1* and *pTKTKK2*, which were constructed by fusing a rice gene *LOC_Os12g16880* or *LOC_Os08g03579* with 16-fold TKTKK coding motifs TKTKKTKTKKKTKKKKKTKKKTKKKTKTKTRS, respectively. Bottom panel showed the binary vector *pTKTKK3*, which was constructed by tandom arraying both *TKTKK1* and *TKTKK2* in inverse order. **(b**,**c)** show the expression patterns of both genes *LOC_Os12g16880* and *LOC_Os08g03579*, respectively. Expression data were achieved from NCBI GEO dataset with accession number GSE21396. Processed signal intensity, which was converted from processed raw data, was used to estimate the expression abundance in each gene among 11 tissues from different developmental stages. The prefix “*LOC_*” in each gene locus name was omitted for convenience. Tissue names in **(b**,**c)** were labelled in each column.

To synthesize a high lysine and threonine coding gene, we selected two endogenous rice genes as templates. We first selected the rice gene *LOC_Os12g16880*, which encodes a seed storage protein with a putative function in impeding the digestion of plant starch and proteins. The lysine-threonine coding motif was designed as ‘TKTKKTKTKKKTKKKKKTKKKTKKKTKTKTRS’ (T, lysine; K, threonine; R, argenine; S, serine). The gene *LOC_Os12g16880* was fused with the fragment encoding 16 times of the lysine-threonine motifs (top panel in Fig. 1a). The synthetic gene was designated as *TKTKK1*, which encodes a 78.7 kDa protein, consisting of 48.1% lysine and 25.0% threonine. The synthetic gene was under the control of 35S promoter and the resulted construct was named as *pTKTKK1*. Similar to *pTKTKK1*, the gene *LOC_Os08g03579* (encoding an unknown protein) was fused with the same lysine-threonine motifs, resulting in another gene designated as *TKTKK2*, which encodes a 68.7 kDa protein, consisting of 55.5% lysine and 28.0% threonine. Accordingly, the resulted construct was named as *pTKTKK2* (middle panel in Fig. 1a). In the third construct *pTKTKK3*, both genes *TKTKK1* and *TKTKK2* were tandemly arrayed under the 35S promoter with opposite orientation (bottom panel in Fig. 1a). We designated these transgenic rice lines generated from these three constructs as *35S::TKTKK1*, *35S::TKTKK2* and *35S::TKTKK3*, respectively.

The gene *LOC_Os12g16880* was mainly expressed in endosperm (Fig. 1b; Supplementary Table S1). Less expression abundance was observed in ovary followed by embryo (Fig. 1b). Very low expression level was detected in the remaining tissues. Thus, we selected the endosperm-preferred gene so that its protein might show more stable storage in seeds as usual. On the contrary, the second gene *LOC_Os08g03579* was totally different from the first one in their expression patterns with very high expression abundance in all tested tissues (Fig. 1c; Supplementary Table S1). As this gene was also highly expressed in endosperm, its protein was supposed to be naturally stored in seeds.

### Molecular characterization of transgenic plants carrying different constructs

**Figure 2:**
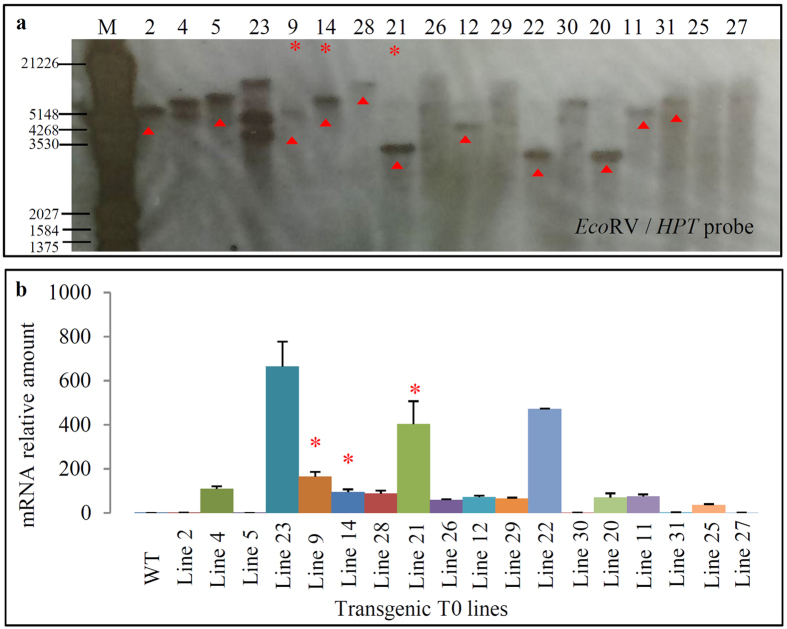
Molecular characterization of *35S::TKTKK1* transgenic plants. **(a)** Copy number detection of T-DNA insertion in transgenic plants by Southern blot hybridization. DNA samples from a total of 18 transgenic plants were restricted by *Eco*RV and then transferred onto nylon membrane for hybridization using the *HPT* probe. The red triangle indicated the lines with single copy of T-DNA insertion. **(b)** Bar diagrams showing expression patterns of the *TKTKK1* gene in the 18 independent transgenic plants by qRT-PCR. The mRNA relative amount (Y axis) was calculated according to the description in Materials and methods. The amplification of an *eEF-1a* gene was used as an internal control to normalize the data. The red arrows in **(a)** indicated the lines with single copy of T-DNA insertion by Southern blotting analysis. The red stars “*” in **(a**, **b)** indicated the lines with single copy of T-DNA insertion and with relatively higher expression level. These three independent lines were selected for further investigation.

We have generated and planted a total of 40 T0 transgenic lines from 60 independent hygromycin resistant calli for the construct *pTKTKK1*. DNA samples extracted from 18 T0 lines were submitted to Southern blot hybridization (Fig. 2a). The blotting data showed that a total of 11 lines might contain single copy of T-DNA insertion as indicated by red triangle. They were lines 2, 5, 9, 14, 28, 21, 12, 22, 20, 11, and 31. The quantitative real-time reverse transcription PCR (qRT-PCR) analysis showed that all of the analyzed lines exhibited at least 1.5-fold higher expression level when compared with wild type (WT) plant (Fig. 2b; Supplementary Table S2). The line 23 showed the highest expression abundance (664.9-fold) but contained three copies of T-DNA insertion (Fig. 2a,b). Finally, we selected three independent lines 9, 14 and 21 for further investigation. These lines showed relatively high expression level for the gene *TKTKK1* and contained single copy of T-DNA insertion.

**Figure 3:**
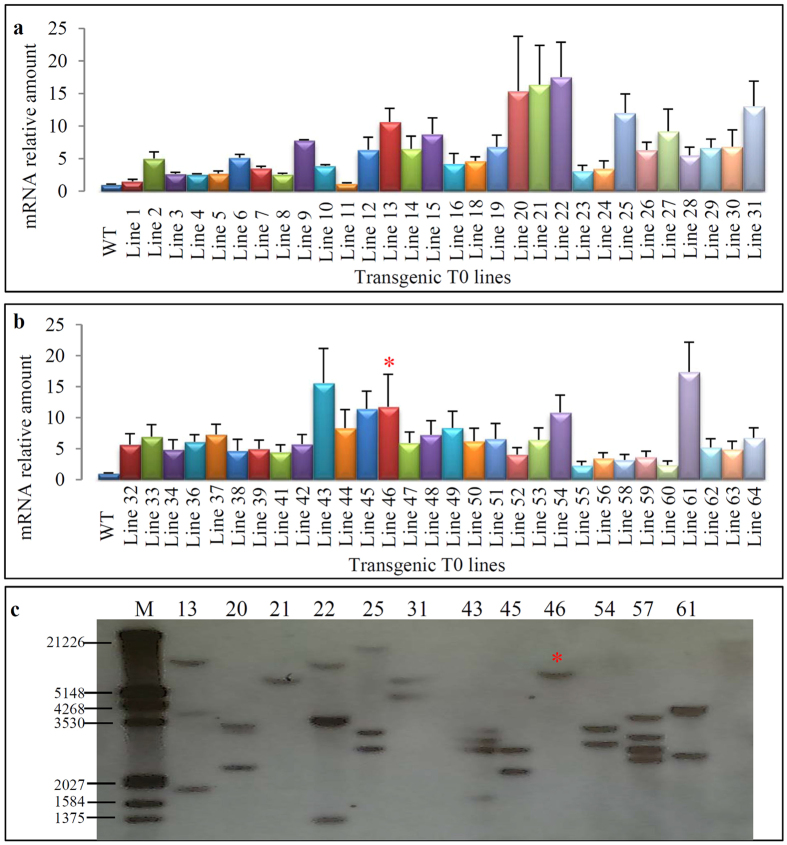
Molecular characterization of *35S::TKTKK2* transgenic plants. **(a,b)** show the expression patterns of the *TKTKK2* gene in 60 transgenic plants by qRT-PCR. The mRNA relative amount (Y axis) was calculated as shown in Materials and methods. The *eEF-1a* gene was used as an internal control to normalize the data as shown in Fig. 2b. (**c**) Copy number detection of T-DNA insertion in transgenic plants by Southern blot hybridization. DNA samples from top 12 transgenic plants in expression were restricted by *Eco*RV and then transferred into nylon membrane for hybridization using the *HPT* probe. The red star “*” in **(b,c)** indicated the line with high level of expression signal and with single copy of T-DNA insertion, which was selected for further analysis.

For the construct *pTKTKK2*, a total of 60 T0 lines were regenerated from 80 independent transgenic calli. Based on the qRT-PCR analysis (Fig. 3a,b; Supplementary Table S3), the *TKTKK2* gene showed higher level of expression in all transgenic lines when compared with its endogenous gene *LOC_Os08g03579*. However, the relative expression abundance was significantly lower than those lines from *pTKTKK1* (Fig. 2b). This should be due to that the expression level of calibrator gene *LOC_Os08g03579* in WT leaves is much higher than that of the gene *LOC_Os12g16880* (Fig. 1b,c). We selected the top 12 lines based on their expression abundance for Southern blot hybridization (Fig. 3c). The analysis showed that most of the lines have 2–4 copies of T-DNA insertion and only two lines (21 and 46) contained single copy of T-DNA integration as indicated by red stars (Fig. 3c), which were selected for further investigation.

**Figure 4:**
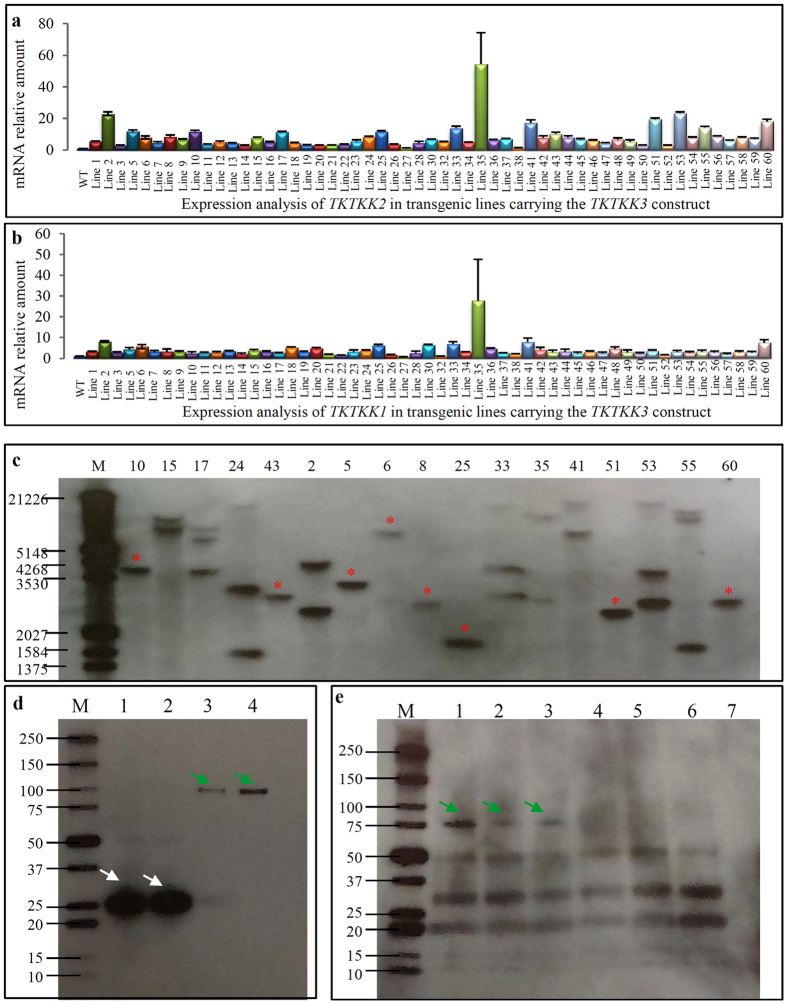
Molecular characterization of *35S::TKTKK3* transgenic plants and western blot hybridization. **(a,b)** show the bar diagrams of expression patterns of both genes *TKTKK1* and *TKTKK2* in 55 *35S::TKTKK3* transgenic plants by qRT-PCR, respectively. **(c)** Southern blot hybridization for detecting copy numbers of T-DNA insertion. DNA samples (line numbers were labelled on the top of the panel) were digested by the restriction enzyme *Eco*RV and were then transferred into nylon member for hybridization with the *HPT* probe. The red stars “*” indicated the lines with single copy number of T-DNA insertion. **(d,e)** Western blot hybridization using crude proteins from *E. coli* **(d)** and T2 rice grains **(e)**, respectively. The “M” in **(d,e)** indicates the protein marker used for the hybridization. The numbers in **(d)** indicate the crude proteins extracted from *E. coli* carrying the plasmid *pGEX-6P-1* (1 and 2) and *pGEX-6P-1::TKTKK1* (3 and 4). The white arrows indicated the GST expression in the *E. coli* carrying the plasmid *pGEX-6P-1*. The green arrows indicated the GST fusion protein expression in the *E. coli* carrying *pGEX-6P-1::TKTKK1*. The numbers in **(e)** indicate the different transgenic lines used for crude proteins extraction from rice seeds. Three lines (1, line 9, 2, line 14 and 3, line 21) were from *35S::TKTKK1*. Line 46 (indicated by 4) and Line 5 (indicated by 5) were from *35S::TKTKK2* and *35S::TKTKK3*, respectively. The number 6 indicates WT and 7 indicates negative control. The green arrows indicated the stable expression of TKTKK1 protein in lines 9, 14 and 21 by Western blot hybridization.

For the construct *pTKTKK3*, we have also generated and planted a total of 60 T0 lines regenerated from 85 independent transgenic calli. As two TKTKK-motif coding genes were overexpressed in these transgenic plants, we measured the expression level of these two genes including *TKTKK1* (Fig. 4a; Supplementary Table S4) and *TKTKK2* (Fig. 4b; Supplementary Table S4). Among the 60 transgenic lines, expression data from 5 of these lines were not qualified for further analysis, thus, only the expression data from the remaining 55 lines were presented in this study. For the *TKTKK1* gene, some of the 55 analyzed lines showed the similar expression level to the endogenous gene *LOC_Os12g16880* (1.29-fold for Line 27) and others showed higher (up to 54.45-fold for Line35) expression level. Similar results were observed for the *TKTKK2* gene (Fig. 4b). Generally, expression level of either *TKTKK1* or *TKTKK2* in 35S::*TKTKK3* transgenic plants was lower than that in 35S::*TKTKK1* or 35S::*TKTKK2* plants. For example, for the *35S::TKTKK1* plants, many lines showed more than 100-fold higher expression than the endogenous gene *LOC_Os12g16880*, significantly higher than that in the *35S::TKTKK3* plants (Figs 2b and 4a,b). The data suggested the slight co-suppression between *TKTKK1* and *TKTKK2* when they were both overexpressed under the control of 35S promoter.

Based on the expression data of *TKTKK1* and *TKTKK2*, we have selected top 17 lines for T-DNA copy number detection by Southern blot hybridization (Fig. 4c). Most of transgenic lines with relatively higher expression level for these two genes contained two or more copies of T-DNA insertion. We have detected a total of 6 lines with single copy of T-DNA insertion and these lines showed similar expression level for both genes *TKTKK1* and *TKTKK2*.

### Synthetic fusion proteins were stably expressed in the *35S::TKTKK1* transgenic plants

As the synthetic genes encode proteins with 16 times of repeated motifs ‘TKTKKTKTKKKTKKKKKTKKKTKKKTKTKTRS’, one might argue whether they could be translated and be stable in rice seeds. We first tested the protein stability in *E. coli* cells. The synthetic gene *TKTKK1* was fused with *GLUTATHIONE S-TRANSFERASE (GST)* by sub-cloning into the *pGEX-6P-1* vector. The resulted plasmid was used for *E. coli* transformation followed by protein extraction and western blot hybridization. The blotting result using GST antibody showed that the fusion protein could be stably expressed in the *E. coli* cells with expected molecular weight (78.7 kDa for TKTKK1 and 28 kDa for GST, Fig. 4d). Furthermore, we also detected the protein stability in transgenic rice seeds by Western blot hybridization. The result showed that the synthetic fusion proteins could be detected in all *35S::TKTKK1* transgenic rice seeds (Fig. 4e). Although mRNA transcript signal could be detected in the *35S::TKTKK2* or *35S::TKTKK3* transgenic plants, very faint signal was detected in *35S::TKTKK2* and no protein signal was detected in *35S::TKTKK3* transgenic seeds (Fig. 4e). The data suggested that the stable expression of synthetic proteins might be dependent on the fused endogenous genes and used constructs.

### Preliminary phenotyping and genotyping in T1 generation of transgenic plants

**Figure 5:**
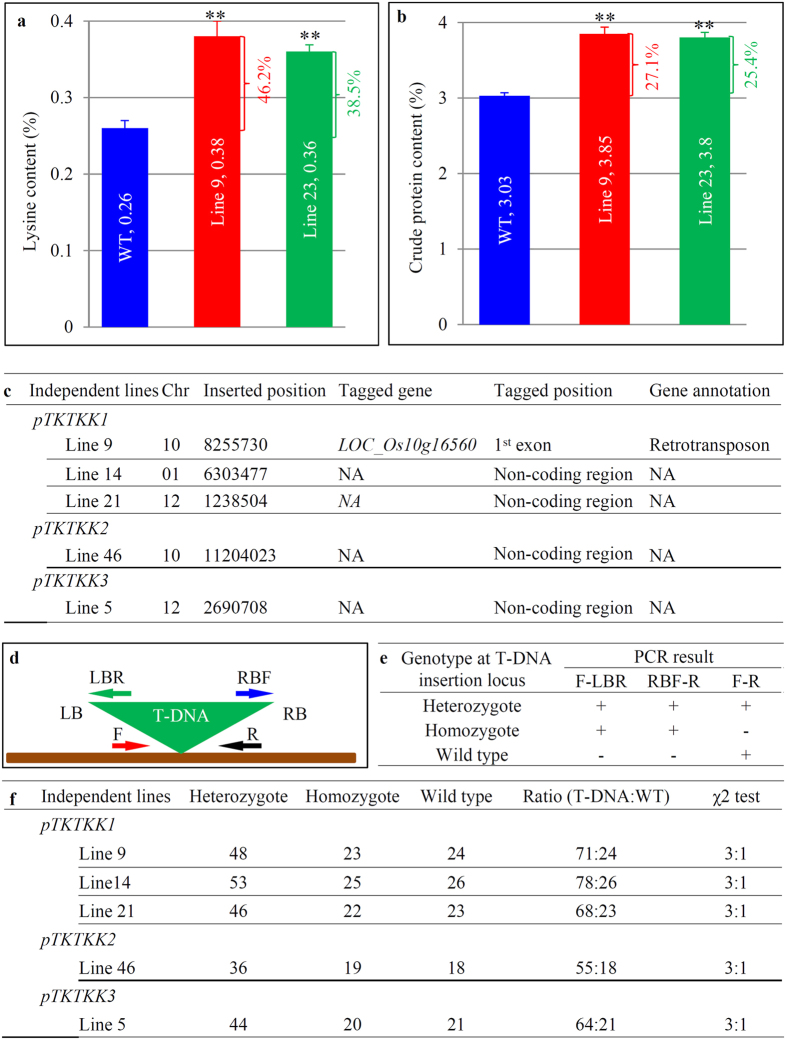
Preliminary analysis of lysine and crude protein and FST analysis. (**a,b**) Lysine and crude protein content. respectively, in T0 generation of transgenic plants carrying the construct *35S::TKTKK1*. **(c)** Characterization of FSTs of T-DNA insertion. A total of 5 independent lines from *35S::TKTKK1*, *35S::TKTKK2* or *35S::TKTKK3* were selected for FST analysis. NA, not available. **(d)** Schematic diagram for PCR genotyping T1 or T2 generation of transgenic plants. Forward (F) and reverse (R) primers were designed according to the FSTs. Left border reverse (LBR) and right border forward (RBF) primers were designed according to T-DNA border sequence. **(e)** Identification of three different genotypes including heterozygotes, homozygotes and WT in T1 or T2 transgenic plants based on PCR results. The symbol “+” indicated that PCR fragment could be amplified using corresponding primer sets and “−” indicated no PCR product. **(f)** Genotyping of five populations from 5 T1 generations of transgenic plants and their χ2 test.

As expression level varied with a large range for the *35S::TKTKK1* transgenic lines, we selected two lines (9 and 23) for testing lysine and protein content. The line 9 showed the middle level of expression abundance with single copy of T-DNA insertion while the line 23 showed the highest expression level with three copies of T-DNA insertion (Fig. 2). Both lines contained significantly higher level of lysine content in two-month-old vegetative stage of plants (Fig. 5a; Supplementary Table S5). The lysine content was increased by 46.2% for line 9 and by 38.5% for line 23. These two lines showed no difference in lysine content by statistical analysis although they exhibited significant difference in their expression level. The crude protein content was enhanced by 27.1% and 25.4% for lines 9 and 23, respectively when compared with the WT plants (Fig. 5b; Supplementary Table S5). Thus, our preliminary data showed that one of our synthetic genes should play a role in improving lysine and protein content.

Thermal asymmetric interlaced PCR (TAIL-PCR)^32^ was employed to amplify T-DNA flanking sequence tags (FSTs) for single copy of T-DNA insertion lines. A total of 5 independent lines were subjected to FST analysis (Fig. 5c). For the *35S::TKTKK1* transgenic line 9, T-DNA was inserted into the 8,255,730^th^ bp of chromosome 10. The T-DNA was inserted into the first exon of the gene *LOC_Os10g16560*, which was annotated to encode a retrotransposon. For line 14, T-DNA was inserted into the 6,303,477^th^ bp of chromosome 1 and no gene was tagged. For line 21, T-DNA was inserted into the 1,238,504^th^ bp of chromosome 12 and no annotated gene was tagged. In the *35S::TKTKK2* line 46, T-DNA was inserted into the 11204023^rd^ bp of chromosome 10 and no gene was tagged by the T-DNA insertion. In the line 5 carrying the construct *pTKTKK3*, the T-DNA was inserted into the 2690708^th^ bp of chromosome 12. Similarly, no gene was tagged as the T-DNA was inserted into the non-coding region. Thus, in these lines, T-DNA was inserted into either non-coding region or a retrotransposon region, which minimized the phenotypic variation from T-DNA mutagenesis.

Based on T-DNA tagging positions in the rice genome, we designed three primer sets to identify the genotypes (homozygote, heterozygote and WT) at T-DNA insertion locus for each line as explained in the figure (Fig. 5d,e). Based on the PCR analysis, the T-DNA locus in each line was segregated at the ratio 3:1 by χ2 test at p < 0.01 for all 5 population from 5 independent lines (Fig. 5f). Thus, seeds were harvested from homozygous transgenic plants for further phenotype investigation. All DNA samples were also subjected to another set of PCR using primers designed from the selection marker gene *HPT* (encoding hygromycin phosphotransferase). The experiment showed that the marker gene was detected in all heterozygotes and homozygotes and no signal was detected in the WT genotype. The data further confirmed that only single copy of T-DNA was integrated into the rice genome in each line.

### Fusing *TKTKK* coding motifs to a rice endogenous gene significantly increased lysine, threonine and crude protein content in rice seeds

Seeds harvested from homozygous T2 transgenic plants in each of 5 lines were subjected to measuring protein content and quality. We have tested lysine, threonine, total amino acids and crude protein content in 3 independent *35S::TKTKK1* lines. For *35S::TKTKK2* lines, only two independent lines with single copy of T-DNA insertion was generated and we selected line 46 for further analysis as the expression level of *TKTKK2* in this line is lower than another one (Fig. 3). For the *35S::TKTKK3* lines, a total of 8 independent lines have single copy of T-DNA insertion and they showed similar expression level for both *TKTKK1* and *TKTKK2* (Fig. 4) and we randomly selected line 5 for further investigation.

**Figure 6:**
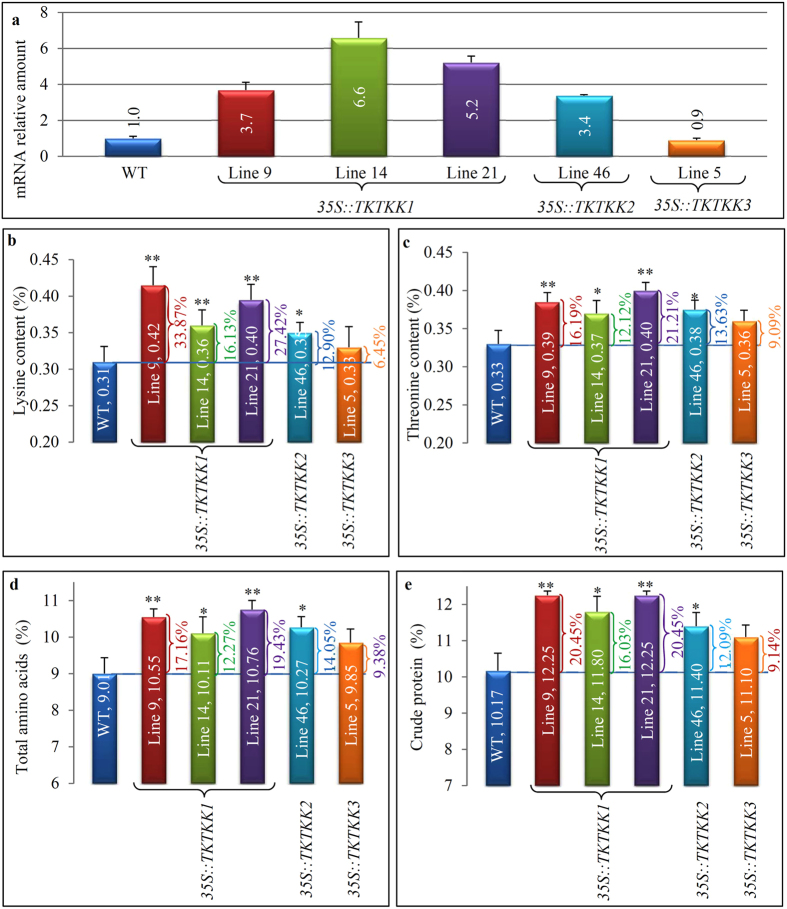
Expression analysis of synthetic genes and measurement of amino acid and crude protein contents in transgenic rice seeds. (**a**) The qRT-PCR analysis of synthetic genes in transgenic seeds from 3 constructs. (**b**–**e**) shows the content (percentage) of lysine (**b**), threonine (**c**), total amino acids (**d**) and crude protein (**e**) in 5 independent transgenic plants. Matured seeds were dried at 37 °C for 3 days and were then subjected to amino acid and protein measurement. Asterisks “*” and “**” indicate significant differences at P < 0.05 and P < 0.01, respectively.

We first analysed the expression abundance of targeted genes in transgenic seeds in these 5 independent lines (Fig. 6a; Supplementary Table S6). The qRT-PCR data showed that four lines including 9, 14 and 21 from *35S::TKTKK1* and 46 from *35S::TKTKK2* exhibited at least 3-fold higher expression level when compared with corresponding endogenous genes (Fig. 6a). However, low expression level was detected for the line 5 from *35S::TKTKK3*. We then further investigated the protein and lysine/threonine content in these lines. Our data showed that lysine content was increased by 16.13–33.87% in 3 independent *35S::TKTKK1* lines when compared with WT, statistically higher than WT (Fig. 6b; Supplementary Table S6). For the *35S::TKTKK2* line 46, 12.90% increase was detected with statistically higher than WT (Fig. 6b). However, for the *35S::TKTKK3* line 5, only 6.45% more lysine content was detected with no statistical difference when compared with WT (Fig. 6b). On the other hand, threonine content was improved by 12.12–21.21% in 3 *35S::TKTKK1* lines when compared with WT (Fig. 6c; Supplementary Table S6). For the *35S::TKTKK2* line 46, threonine was enhanced by 13.63%, significantly higher than WT (Fig. 6c). In the *35S::TKTKK3* line 5, only 9.09% increase in threonine content was measured, with no statistical difference when compared with WT (Fig. 6c). For total amino acid analyses, three lines from *35S::TKTKK1* showed 17.16–19.43% increase, significantly higher than WT (Fig. 6d; Supplementary Table S6). For the *35S::TKTKK2* line 46, its total amino acid content showed up to 14.05% increase and is statistically higher than WT. However, the *35S::TKTKK3* line 5 showed no significant difference although its content was increased by 9.38% (Fig. 6d). We then compared the crude protein content of all these 5 independent transgenic seeds with that in WT seeds (Fig. 6e; Supplementary Table S6). Similarly, in all three *35S::TKTKK1* lines, protein content was increased by 16.03–20.45%. The *35S::TKTKK2* line 46 showed significant increase by 12.09% and another line from *35S::TKTKK3* showed no significant difference. In general, both *35S::TKTKK1* and *35S:TKTKK2* significantly increased the content of lysine, threonine, total amino acids and crude protein while *35S::TKTKK3* showed limited improvement in protein content and quality.

### Overexpression of *TKTKK1* or *TKTKK2* might not affect normal growth and development in rice

**Figure 7:**
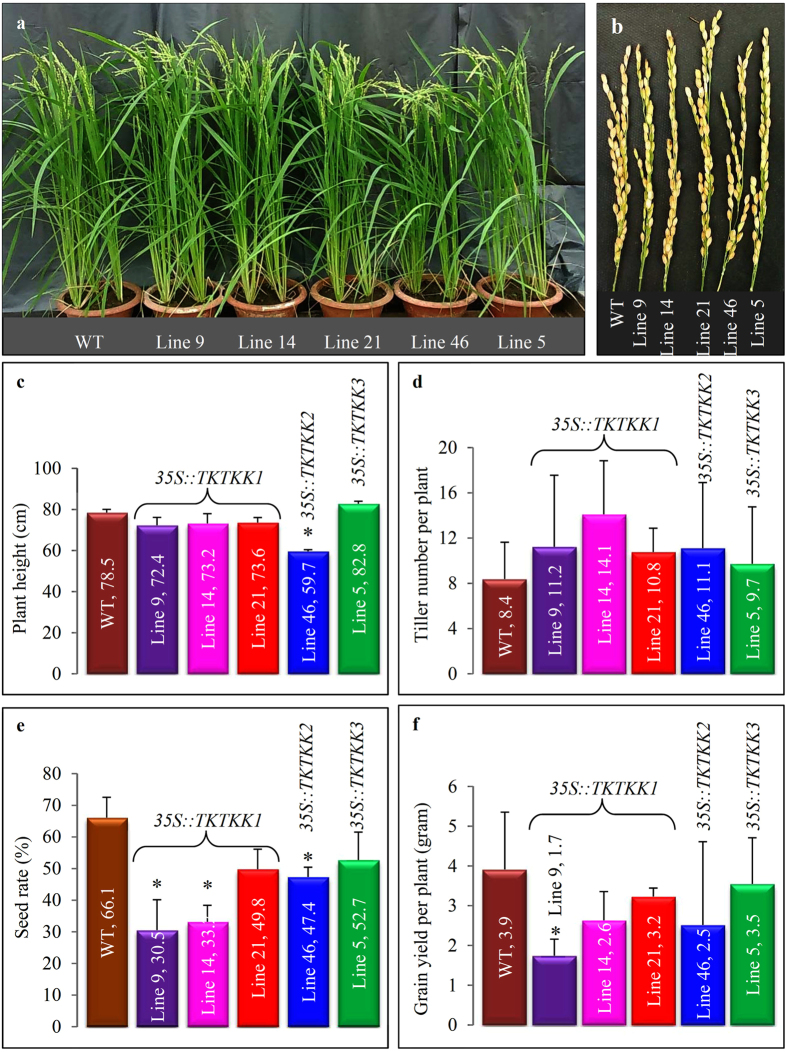
Investigation on agronomical traits in transgenic plants. **(a)** Phenotypic characterization of rice plants at the grain filling stage in WT and 5 independent transgenic lines. **(b)** Panicles at the mature stage in WT and 5 independent transgenic lines. **(c–f)** shows average plant height (cm), average tiller numbers per plant, average seeding rate, and average grain yield per plant, respectively, in WT and 5 independent transgenic lines. Only the productive tillers that could produce spikes and seeds were scored in this investigation. The seeding rate was determined using the ratio of normally filled seeds among total panicle grains. In **(a–f)**, lines 9, 14 and 21 carried the *35S::TKTKK1* construct; line 46 and line 5 were from the constructs *35S::TKTKK2* and 35S::*TKTKK3*, respectively.

Generally, the line 46 from *35S::TKTKK2* exhibited shorter height when compared with the remaining plants (Fig. 7a). At the mature stage, normally filled grains (full seeds) were less in lines 9 and 14 (Fig. 7b). Detail measurement showed that all three transgenic plants from *35S::TKTKK1* showed similar height to WT plants (Fig. 7c; Supplementary Table S7). Similar result was observed in *35S::TKTKK3* (Fig. 7c). However, the line 46 from *35S::TKTKK2* showed shorter height when compared with WT plants (Fig. 7c). We calculated till number per plant in both WT and transgenic plants and found that no significant difference was observed when compared with WT plants (Fig. 7d; Supplementary Table S7). We then surveyed the average seeding rate and showed that both line 21 from *35S::TKTKK1* and line 5 from *35S::TKTKK3* exhibited similar seeding rate to that in WT plants (Fig. 7e; Supplementary Table S7). However, the remaining 3 plants from either *35S::TKTKK1* or *35S::TKTKK2* showed significant lower seeding rate when compared with WT plants (Fig. 7e). Finally, we measured the average grain yield per plant and found that only the line 9 showed lower grain yield per plant (Fig. 7f; Supplementary Table S7). The remaining 4 transgenic lines showed no significant difference when compared with WT plants (Fig. 7f). In this study we have generated at least one transgenic plant (line 21), which showed normal growth and development but improved protein quality and quantity. All in all, although some transgenic plants showed shorter plant height, lower seeding rate or grain yield, these changed phenotypes were not related to the overexpression of any of synthetic fusion genes (Fig. 7).

## Discussion

Generally, content of essential amino acids in an organism could be improved by increasing either protein-bound or free amino acids. Although free essential amino acids could be increased by around 60-fold in rice seeds^8^, free amino acids pool is small compared to the protein-bound amino acids^33^ and thus, limiting the net accumulation of essential amino acids. Additionally, free lysine is known to react on heating with sugars to form chemical compounds called Nε-(Carboxymethyl) lysine, which is one of well-characterized advanced glycation end products (AGEs)^31^. AGEs are a diverse group of highly oxidant compounds that are linked to numerous diseases, including diabetes, Alzheimer’s disease and cancers^34^,^35^. The transgenic maize variety LY038 with 50-fold higher levels of free lysine in the maize kernel by expressing the bacterial *DHPS* has been approved for animal food use in Japan, S. Korea, Canada, Australia, New Zealand and the US (https://www.isaaa.org/gmapprovaldatabase/event/default.asp?EventID=146). However, Monsanto has withdrawn its application due to its potential food safety (http://www.independentsciencenews.org/news/transgenic-corn-ly038-withdrawn/). All these data imply the importance for us to further improve the protein-bound essential amino acids. However, currently employed strategies to improve protein-bound essential amino acids have various disadvantages such as protein instability for synthetic genes, protein allergy in seeds, and other accompanied agronomic traits including low seeding set, low yield etc. In this study, we used the rice endogenous genes as templates to design new genes by fusing *TKTKK* coding motifs to further improve both lysine and threonine content. Our data showed that the expressed proteins could stably exist in either *E. coli* cells or transgenic rice seeds (Fig. 4d,e). Expression of such fusion proteins has limited effect on other agronomic traits and our data showed no relationship between changed agronomic traits and T-DNA integration (Fig. 7). Thus, our study provides an alternative strategy to further improve protein-bound essential amino acids.

Artificially modified genes have been used to improve protein quality as such a strategy is a straightforward molecular improvement of amino acid constituent^36^. Many genes have been modified including these genes encoding α-zein, γ-zein, β-phaseolin, 2S albumin, Braizil nut 2S etc^36^,^37^. However, major challenge to this strategy is the instability of modified proteins^36^,^37^. In this study, the original protein sequences were not modified and were fused with lysine- and threonine-rich motifs. Thus, the employed strategy might minimize the change in protein stability. On the other hand, some of seed proteins are responsible for allergy. We submitted both genes *Os12g16880* and *Os08g03579* as well as TKTKK motif sequences for BLAST searches against the allergy database (http://www.allergenonline.org/index.shtml). The results showed that no sequence homology was found in the database. We have also used AllerHunter (http://tiger.dbs.nus.edu.sg/AllerHunter/running.html) and Allerdictor (http://allerdictor.vbi.vt.edu/predict/) for allergen prediction of TKTKK1 and TKTKK2. No potential allergen was predicted. Thus, both employed rice genes and the designed TKTKK motifs could be used to synthesize high lysine/threonine coding genes without potential risk for the production of allergy proteins.

The application of synthetic biology on improving protein quality has been carried out long time ago. For example, several genes have been designed according to an alpha-helical coiled-coil structure and these genes encoded high lysine proteins^26^. As a result, lysine content in seeds was increased in these transgenic tobacco seeds carrying one of these genes. However, lysine content was increased by less than 20%^26^, which might be due to that these genes encoded high lysine proteins with very small molecular weight (3–7 kDa). In this study, lysine/threonine-rich fragments consist of 73% and 83% of total amino acids with molecular weight at 78.7 kDa and 68.7 kDa in both *TKTKK1* and *TKTKK2* genes, respectively. These synthetic genes encode proteins with similar structure, which forms alpha-helixes followed by coiled-coil tails (Supplementary Figure S1). Although the predicted structures are similar for these two proteins, TKTKK1 showed more stability and contributed more efficiently to the improvement of protein quantity (Fig. 6). Thus, more experiments should be carried out to test which endogenous genes should be employed to form TKTKK fusion proteins to synthesize stable proteins. Currently, we fused 16 times of TKTKKTKTKKKTKKKKKTKKKTKKKTKTKTRS motifs to an endogenous rice protein. We may also need to figure out the optimized motif numbers to achieve the highest level of protein improvement.

Evidence has shown that over-expression of high lysine coding genes could significantly increase lysine content in transgenic plants^21^,^23^,^38^,^39^. Similarly, over-expression of genes encoding proteins with higher percentage of threonine or other amino acids could also increase the level of threonine or other amino acids^7^,^40^,^41^,^42^. However, in plants, limited genes are available that encode high lysine or threonine proteins. One of widely employed high lysine coding genes is *SB401* from maize, which encodes a protein consisting of 16.7% of lysine^38^. In rice, our genome-wide survey showed that only 11 genes encoded proteins (with >150 amino acid long) containing more than 20% lysine. However, these protein sequences consist of only 0.72–7.89% threonine. On the other hand, we have detected only 8 genes encoding proteins (no less than 150 amino acids) with more than 15% threonine. No gene encodes a protein with more than 15% lysine and 15% threonine in the rice genome. Thus, high lysine and/or threonine genes should be artificially modified or synthesized so that the lysine /threonine content could occupy higher percentage. However, even in artificially modified genes, the percentage of lysine or threonine among the total amino acids was still not high due to the instability of the modified proteins. For example, in the synthetic gene *CP 3-5*, it encodes only 31% lysine and 20% methionine^26^. In this study, we increased lysine and threonine percentages to 48.1% and 25.0%, respectively, by fusing a TKTKK coding motifs to endogenous rice genes. The synthetic genes could be stably expressed in the rice genome and generated stable proteins in rice seeds as detected by Western blot hybridization (Fig. 4e). As a result, we successfully generated high lysine plants and seeds. We have over-expressed two genes encoding proteins with molecular weight at 78.7 and 68.7 kDa, respectively. More experiments should be carried out to demonstrate whether the molecular weight or motif length of synthetic proteins might affect the protein stability or lysine/threonine content in transgenic plants or seeds.

Although the efficient methods to increase free lysine or threonine through metabolic pathway have been reported, the strategies might not be commercially used to improve essential amino acid content due to (1) low total free amino acid content compared with protein-bound amino acids and (2) possible healthy risk from free amino acids. Thus, further improvement of protein-bound essential amino acids has been put in the first choice. Our study showed that artificially fusing TKTKK coding sequences to an endogenous rice gene and then over-expressing it in the rice genome could significantly increase both crude protein and essential amino acid content. Our research might provide an alternative way to improve protein content and quantity. Our data showed that employed rice genes might also affected the accumulation of TKTKK-fused protein. Therefore, higher accumulation of the fused protein through this strategy should be achieved by selecting and optimizing the endogenous rice genes which were used to fuse with TKTKK motif coding sequences. Currently, we have tested only two rice genes *LOC_Os08g03579* and *LOC_12g16880*. These two genes showed very high expression level in the rice plants (Fig. 1). As a result, this might affect the expression level of *TKTKK1* or *TKTKK2* and subsequent accumulation of their fused proteins. Therefore, selection of seed-specific genes with low expression level should be an alternative way to further improve lysine and threonine content through fusing with TKTKK coding sequences.

Increase of free lysine content has been achieved by transgenic expression of bacterial lysine feedback-insensitive *DHPS* genes and this method has been used for many species as shown in the Introduction. However, some limitations were found in maize^14^,^15^,^16^, rice and barley^17^,^18^. Thus, it is still not a universal method. On the other hand, modified high lysine genes were used for lysine improvement in limited species. For example, a modified gene encoding a lysine-rich zein was expressed normally in maize^39^. However, the modified protein was abnormally localized on cell wall instead of endoplasmic reticulum^43^. Current data showed that no universal method can be used for improving amino acid content. We have developed an efficient strategy to improve lysine, threonine and crude protein content in rice. Two genes have been used to fuse with TKTKK coding motifs for improving protein content and quality. However, the seed-specific gene *LOC_Os12g16880* showed higher efficiency. Thus, in order for us to apply this method to other species, seed-specific endogenous genes in targeted species might be selected to fuse with the TKTKK coding motifs for transgenic expression. As we used endogenous genes for fusion expression and their proteins should be more stable, thus, providing a universal strategy for improving lysine, threonine and crude protein content in any crop species and even in vegetable plants.

## Methods

### Plant materials and growth conditions

Rice variety Nipponbare (*Oryza sativa*) was used for all experiments. Rice seeds were germinated and were then transferred into soil pots. Plants were grown in greenhouse under natural sunlight and temperature conditions. The mature seeds were also used to induce calli for *Agrobacterium*-mediated genetic transformation.

### Selection of candidate endogenous genes and expression analysis of both *LOC_Os12g16880* and *LOC_Os08g03579*

The selection standard of endogenous genes for fusing expression with TKTKK motifs is based on gene size, expression and their putative functions. We selected these genes encoding proteins with 100–200 amino acids long so that the fused proteins are no more than 100 KDa in molecular weight. We have identified 12,978 annotated genes encoding such length of proteins based on the Rice Genome Annotation Project database. We surveyed the expression profiling of these genes based on the microarray dataset with the NCBI GEO accession number GSE21396. Finally, we selected one gene *LOC_Os12g16880*, which showed seed-specific expression and encodes a seed storage protein. Another gene *LOC_Os08g03579* showed high expression abundance in multiple tissues including seeds and encodes an expressed protein with unknown function, which might reduce the negative effect of this gene on plant growth.

### Cloning of candidate genes, vector construction and plant transformation

Total RNA samples from 14-day-old leaves were prepared using Qiagen total RNA Extraction Kit. Total RNA samples from mature seeds were isolated using the method as described by Wang *et al*.^44^. Coding regions of *LOC_Os12g16880* and *LOC_Os08g03579* were amplified by RT-PCR using the primer sets as listed in Supplementary Table S8. RT-PCR was carried out using Qiagen One-step Kit according to the manufacture’s instruction.

Two 99-bp single strain oligo fragments ACCAAGACGAAGAAGACGAAGACCAAGAAGAAGACCAAGAAGAAGAAGAAGACGAAGAAGAAGACCAAGAAGAAGACCAAGAAGACGAAGACCACGAAG and TGCTTCTGGTGCTTCTTCTTCTGCTTCTTCTTCTGGTTCTTCTTCTTCTTCTTCTGGTTCTTCTTCTGGTTCTTCTGCTTCTTCTGGTTCTGCTTCTGC were ordered from the Integrated DNA Technologies (http://www.idt.com). They were complementary each other and were mixed together as DNA templates for PCR amplification using the primer set ggatccACCAAGACGAAGAAGACGAAGA and agatctCGTCTTCGTCTTGGTCTTCTTC by introducing both restriction enzymes *Bam*HI and *Bgl*II sites as underlined in the primer sequences. The PCR fragment was purified from Agarose gel and then sub-cloned into pGEM-T Easy vector (Promega). The *Bam*HI and *Bgl*II restriction fragments were ligated each other and were then sub-cloned into the pGEM-T Easy vector again to get the 2 times of the STKTKKTKTKKKTKKKKKTKKKTKKKTKKTKTTKRS coding sequence. The procedure was repeated again and again and finally resulted in a 16 times of the STKTKKTKTKKKTKKKKKTKKKTKKKTKKTKTTKRS coding sequence. The coding sequence was then fused with the 3′ end of an endogenous gene either *LOC_Os12g16880* or *LOC_Os08g03579*. The fused genes were sub-cloned into pCAMBIA1300 Ti-derived binary vector (CAMBIA, Canberra, Australia; http://www.cambia.org.au) under the control of 35S promoter.

All the three constructs were transformed into *Agrobacterium tumefaciens AGL 1* by electroporation using GIBCO-BRL Cell-Porator. Rice callus induction, *Agrobacterium*-mediated transformation, marker selection and resistant callus regeneration were performed as previously described^45^.

### T-DNA copy number detection by Southern blot hybridization

A total of six micrograms of genomic DNA in each line were digested by restriction enzyme *Eco*RV and was then separated by 0.7% agarose gels. The separated DNA samples were then transferred onto nylon membranes for Southern blot hybridization. The probe was prepared from the *HPT* gene and was labelled with DIG Probe Synthesis Kit (Roche), using the primer set listed in Supplementary Table S8. DNA blots were hybridized with the DIG-labelled probe in DIG easy Hyb solution (Roche Applied Science, Mannheim, Germany) at 42 °C. Detection was carried out according to manufacturer’s protocol using DIG Wash and block Buffer set and chemiluminescent substrate CDP-Star^TM^ (Roche).

### Expression analysis of targeted genes in transgenic plants by qRT-PCR

For qRT-PCR analysis, three biological replicates were carried out and triplicate quantitative assays for each replicate were performed using the AB power SYBR Green PCR Master mix kit (Applied Biosystems, P/N 4367659) according to the manufacturer’s protocol. The qRT-PCR reactions were performed using Applied Biosystems (AB) 7900HT Fast Real-Time PCR system 384 well formats. The amplification of an *eEF-1a* gene was used as an internal control to normalize the data and corresponding sequences of these primers were listed in Supplemental Table S1. The ∆CT and ∆∆CT were calculated according to our previous description^46^. The mRNA relative amount was estimated as 2^−∆∆CT^, which was used for all chart preparations.

### Amplification of T-DNA FSTs and genotyping of T1 transgenic plants

TAIL-PCR^32^ was carried out to amplify the sequence tags flanking the T-DNA insertion. The obtained FSTs were subjected to BLASTN searches to locate the position of T-DNA insertions. The Rice Genome Annotation Project database (http://rice.plantbiology.msu.edu/index.shtml) was used to annotate the tagged genes or chromosomal positions. Based on the FSTs, three pairs of primer sets were designed as described in Fig. 5d to differentiate heterozygotes, homozygotes and WT. All primer sequences were listed in Supplementary Table S8.

### GST-tagged *TKTKK1* construction and Western blot hybridization

The *GST*-tagged *pGEX-6P-1* vector (GE Healthcare Life Sciences) was used for sub-cloning *TKTKK1* by fusing with the GST sequence at its 3′-terminal. After verification by sequencing, the new plasmid pGEX-6P-1 with *GST-TKTKK1* was transformed into the *E. coli* BL21. A total of 500 mg of rice seed power in each sample was used for protein extraction and the resulted supernatant was transferred into SnakeSkin^TM^ Dialysis Tubing (10K MWCO, 22 mm, ThermoFisher Scientific) for dialysis against PBS buffer (change buffer 8–12 hours) at the chill room for 2 days. Crude proteins were separated on the mini-protein precast gel (Bio-Rad) and were then transferred onto nitrocellulose membrane.

For detecting protein expression in the *E. coli* system, GST (1E5) mouse monoclonal, SC-53909 from Santa Cruz Biotechnology was used as the primary antibody. The anti-mouse IgG HRP from GE Healthcare Life Sciences was used as the secondary antibody. For detecting the protein expression in the rice seeds, the 14-aa peptide KKKTKTKTRSTKTK specific to the synthetic genes was used as antigen for antibody synthesis by GenScript, Piscataway, NJ. The HRP- Goat-Rabbit IgG (H+L) DS Grd (from Life technologies) was used as the secondary antibody. Western blot hybridization was carried out using Bio-Rad's Western blotting systems according to the manufacturer’s instructions.

### Measurement of amino acids and crude protein

High-performance liquid chromatography (HPLC) was used to determine the content of 17 amino acids. Both 2-month-old fresh plants at vegetative stage and mature seeds were submitted to measure the content of amino acids and crude protein. The measurement was carried out by Agri-Food & Veterinary Authority of Singapore (http://www.ava.gov.sg/).

### Investigation of morphological traits of transgenic plants

Besides the measurement of amino acids and crude protein in vegetative stage of plants and mature grains, we have also investigated the morphological traits of transgenic plants by comparing with WT plants. Plant height, tiller number, seeding rate and grain yield were evaluated according to the standard evaluation system for rice (IRRI, 2002). A total of 5 independent homozygous T3 lines were used for the trait investigation. Around 40 individuals in each line were planted in each replicate for all of the survey of morphological traits. Three biological replicates were carried out and the difference was tested by statistical analysis.

## Additional Information

**How to cite this article**: Jiang, S.-Y. *et al.* Improving protein content and quality by over-expressing artificially synthetic fusion proteins with high lysine and threonine constituent in rice plants. *Sci. Rep.* **6**, 34427; doi: 10.1038/srep34427 (2016).

## Supplementary Material

Supplementary Information
