# Supplementary material for: Improving protein content and quality by over-expressing artificially synthetic fusion proteins with high lysine and threonine constituent in rice plants
Source: Sci Rep. 2016 Sep 28;6:34427. doi: 10.1038/srep34427 (PMC5039639; doi:10.1038/srep34427)
Supplement: Supplementary Information [file srep34427-s1.pdf]

Improving protein content and quality by over-expressing artificially synthetic fusion proteins with high lysine and threonine constituent in rice plants

Shu-Ye Jiang, Ali Ma, Lifen Xie and Srinivasan Ramachandran*

Rice Functional Genomics Group, Temasek Life Sciences Laboratory, 1 Research Link, National University of Singapore, Singapore 117604

***Corresponding author:** Srinivasan Ramachandran (sri@tll.org.sg)

Supplementary information

Supplementary Figure S1. Prediction of secondary structures of proteins encoded by two newly synthetic genes

Supplementary Table S1. The average and error bar values in Figure 1

Supplementary Table S2. The average and error bar values in Figure 2

Supplementary Table S3. The average and error bar values in Figure 3

Supplementary Table S4. The average and error bar values in Figure 4

Supplementary Table S5. The average and error bar values in Figure 5

Supplementary Table S6. The average and error bar values in Figure 6

Supplementary Table S7. The average and error bar values in Figure 7

Supplementary Table S8. Primer sequences used in this study

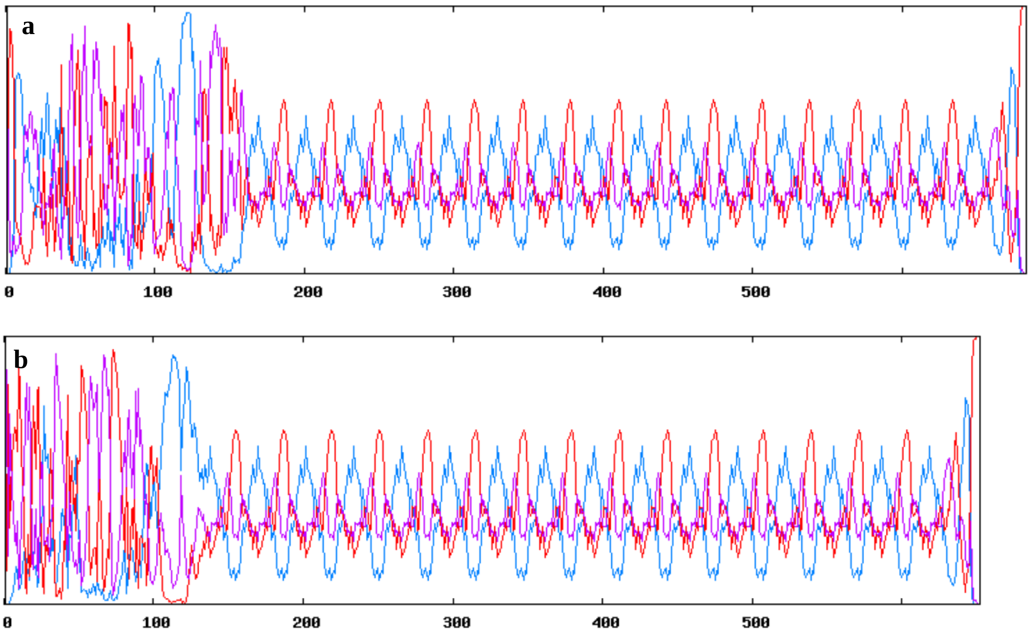

Supplementary Figure S1. Prediction of secondary structures of proteins encoded by two newly synthetic genes. **(a)** Structure of TKTKK1. **(b)** structure of TKTKK2. The secondary structures were predicted by submitting both protein sequences to the prediction database (https://npsa-prabi.ibcp.fr/; Trends Biochem Sci. 2000, 25: 147-150). In both **(a)** and **(b)**, blue, red and orange curves indicated alpha helix, extended strand and random coil structures, respectively.

Supplementary Table S1. The average and error bar values in Figure 1

Tissues	Average value in Figure 1b	Error bar in Figure 1b	Average value in Figure 1c	Error bar in Figure 1c
Leaf blade	5.55	1.81	65779.88	1.81
Leaf sheath	8.34	7.62	110357.28	7.62
Root	50.34	45.95	98709.58	45.95
Stem	6.30	2.77	125638.06	2.77
Inflorescence	8.95	3.80	176092.00	3.80
Anther	88.22	12.63	122933.98	12.63
Pistil	57.25	3.83	148411.83	3.83
Lemma, Palea	44.18	4.32	131738.25	4.32
Ovary	30566.01	3444.37	180516.80	3444.37
Embryo	10683.40	1170.51	195853.20	1170.51
Endosperm	136598.20	3109.17	158751.46	3109.17

Supplementary Table S2. The average and error bar values in Figure 2

Genotype	Average value in Figure 2b	Error bar in Figure 2b
WT	1.00	0.04
Line 2	1.51	0.07
Line 4	109.12	12.81
Line 5	0.51	0.05
Line 23	664.91	112.82
Line 9	166.07	20.14
Line 14	95.33	12.17
Line 28	89.17	12.34
Line 21	402.91	104.34
Line 26	59.61	3.31
Line 12	72.54	5.72
Line 29	65.02	4.94
Line 22	471.91	1.48
Line 30	1.08	0.12
Line 20	70.27	19.09
Line 11	75.22	9.48
Line 31	2.55	0.63
Line 25	36.21	3.42
Line 27	1.55	0.08

Supplementary Table S3. The average and error bar values in Figure 3

Genotype	Average value in Figure 3	Error bar in Figure 3
WT	1.00	0.08
Line 1	1.52	0.31
Line 2	5.01	1.03
Line 3	2.64	0.25
Line 4	2.57	0.10
Line 5	2.71	0.38
Line 6	5.13	0.51
Line 7	3.53	0.31
Line 8	2.57	0.18
Line 9	7.81	0.09
Line 10	3.88	0.18
Line 11	1.14	0.14
Line 12	6.35	1.93
Line 13	10.64	2.08
Line 14	6.50	1.95
Line 15	8.76	2.50
Line 16	4.20	1.59
Line 18	4.64	0.63
Line 19	6.83	1.77
Line 20	15.35	8.45
Line 21	16.37	6.04
Line 22	17.52	5.37
Line 23	3.09	0.87
Line 24	3.47	1.19
Line 25	12.02	2.93
Line 26	6.31	1.20
Line 27	9.20	3.41
Line 28	5.51	1.25
Line 29	6.66	1.34
Line 30	6.84	2.59
Line 31	13.06	3.85
Line 32	5.67	1.74
Line 33	6.95	1.94
Line 34	4.87	1.59
Line 36	6.11	1.15
Line 37	7.27	1.66
Line 38	4.66	1.86
Line 39	4.96	1.43
Line 41	4.45	1.17
Line 42	5.73	1.55
Line 43	15.57	5.58
Line 44	8.33	2.96
Line 45	11.44	2.84
Line 46	11.74	5.25
Line 47	5.92	1.77
Line 48	7.25	2.26
Line 49	8.37	2.68
Line 50	6.22	2.09
Line 51	6.58	2.50
Line 52	4.05	1.13
Line 53	6.43	1.92
Line 54	10.83	2.80
Line 55	2.29	0.64
Line 56	3.45	0.91
Line 58	3.17	0.89
Line 59	3.65	0.94
Line 60	2.36	0.67
Line 61	17.36	4.81
Line 62	5.19	1.41
Line 63	4.91	1.30
Line 64	6.75	1.62

Supplementary Table S4. The average and error bar values in Figure 4

Genotype	Average value in Figure 4a	Error bar in Figure 4a	Average value in Figure 4b	Error bar in Figure 4b
WT	1.00	0.03	1.00	0.08
Line1	5.54	0.39	3.29	0.20
Line2	22.92	9.53	8.14	2.96
Line3	3.02	0.40	2.80	0.32
Line5	11.80	0.64	4.31	0.91
Line6	7.96	0.50	5.68	1.93
Line7	4.63	0.91	3.24	0.56
Line8	8.44	1.10	3.33	1.19
Line9	6.51	1.96	3.08	0.58
Line10	11.68	1.79	2.37	0.81
Line11	3.54	0.18	2.68	0.42
Line12	5.14	0.37	2.71	0.53
Line13	4.36	0.21	3.51	0.25
Line14	2.56	0.63	2.05	0.55
Line15	7.62	0.65	3.73	0.56
Line16	4.97	0.27	3.18	0.48
Line17	11.59	0.26	2.59	0.24
Line18	4.50	0.25	5.23	0.34
Line19	3.09	0.14	3.04	0.41
Line20	2.88	0.32	4.80	0.39
Line21	3.18	0.60	1.84	0.16
Line22	3.57	0.18	1.58	0.13
Line23	5.53	1.16	3.19	0.87
Line24	8.33	0.87	3.67	0.46
Line25	11.68	0.80	6.20	0.51
Line26	3.56	0.79	1.69	0.18
Line27	1.29	0.56	0.71	0.05
Line28	4.50	1.87	2.53	0.14
Line30	6.66	0.11	6.40	0.29
Line32	5.31	0.38	1.01	0.04
Line33	13.86	0.80	6.92	1.22
Line34	4.92	0.36	3.05	0.16
Line35	54.45	38.08	27.88	19.88
Line36	6.46	0.73	4.67	0.33
Line37	7.05	2.15	2.50	0.26
Line38	1.34	0.06	2.11	0.13
Line41	17.25	2.70	7.91	1.87
Line42	7.75	1.53	4.09	1.23
Line43	10.28	1.86	3.00	1.01
Line44	7.99	1.07	3.28	1.09
Line45	6.55	1.52	2.38	0.68
Line46	6.04	0.21	3.11	0.49
Line47	4.20	0.56	2.52	0.49
Line48	6.68	0.70	4.65	0.94
Line49	5.26	2.40	2.93	1.16
Line50	2.84	0.33	2.27	0.49
Line51	19.60	3.09	3.58	0.54
Line52	2.90	0.31	1.47	0.27
Line53	23.37	10.45	3.17	0.73
Line54	7.78	0.89	2.69	0.55
Line55	14.26	1.54	3.26	0.71
Line56	8.51	0.90	2.90	0.59
Line57	5.84	0.92	1.88	0.55
Line58	7.62	1.00	2.98	0.64
Line59	6.97	0.57	2.78	0.57
Line60	18.14	1.51	7.68	1.36

Supplementary Table S5. The average and error bar values in Figure 5

Genotype	Average value in Figure 5a	Error bar in Figure 5a	Average value in Figure 5b	Error bar in Figure 5b
WT	0.26	0.01	3.03	0.04
Line 9	0.38	0.02	3.85	0.09
Line 23	0.36	0.01	3.8	0.07

Supplementary Table S6. The average and error bar values in Figure 6

Figure panel	Genotype	Average	Error bar value
Figure 6a	WT	1.00	0.12
	Line 9	3.69	0.43
	Line 14	6.59	0.88
	Line 21	5.22	0.35
	Line 46	3.38	0.05
	Line 5	0.90	0.11
Figure 6b	WT	0.31	0.02
	Line 9	0.42	0.03
	Line 14	0.36	0.03
	Line 21	0.40	0.02
	Line 46	0.33	0.01
	Line 5	0.34	0.03
Figure 6c	WT	0.33	0.02
	Line 9	0.39	0.01
	Line 14	0.37	0.02
	Line 21	0.40	0.01
	Line 46	0.38	0.01
	Line 5	0.36	0.01
Figure 6d	WT	9.01	0.43
	Line 9	10.55	0.22
	Line 14	10.11	0.45
	Line 21	10.76	0.25
	Line 46	10.27	0.29
	Line 5	9.85	0.37
Figure 6e	WT	10.17	0.49
	Line 9	12.25	0.12
	Line 14	11.80	0.42
	Line 21	12.25	0.12
	Line 46	11.40	0.38
	Line 5	11.10	0.33

Supplementary Table S7. The average and error bar values in Figure 7

Figure panel	Genotype	Plant height	Error bar value
Figure 7c	WT	78.52	0.54
	Line 9	72.35	0.78
	Line 14	73.18	4.78
	Line 21	73.58	2.47
	Line 46	59.65	0.07
	Line 5	82.78333333	1.24922198
Figure 7d	Genotype	Tiller numbers per plant	Error bar value
	WT	8.377777778	3.253943476
	Line 9	11.23450292	6.32570672
	Line 14	14.11190476	4.725675875
	Line 21	10.77318008	2.103639629
	Line 46	11.11111111	5.807785324
	Line 5	9.727969349	5.032348434
Figure 7e	Genotype	Seeding rate	Error bar value
	WT	66.11914446	6.400996472
	Line 9	30.53737923	9.580879196
	Line 14	33.19147688	5.170506337
	Line 21	49.80914633	6.289409637
	Line 46	47.35363428	3.067690795
	Line 5	52.71515646	8.825728219
Figure 7f	Genotype	Grain yield per plant	Error bar value
	WT	3.914444444	1.43989727
	Line 9	1.744532164	0.413932342
	Line 14	2.633531746	0.721604522
	Line 21	3.229632184	0.21457196
	Line 46	2.517111111	2.09353994
	Line 5	3.548291188	1.160821955

Supplementary Table S8: Primer sequences used in this study

Primer Name	Forward primer	Reverse primer	Length (bp)
HPT	CAACCAAGCTCTGATAGAGT	GAAGAATCTCGTGCTTTCAG	745
LOC_Os12g16880	ATGAAGATCATTTTCTTCTTTGCTCTCC	ggatccGTACCAGATACCACCGACCGTG	474
LOC_Os08g03579	ATGTCTTCGGCTGCCAAGCTCG	ggatccACGGTAGGTAAACATTAGCAGC	204
eEF-1a	TTTCACTCTTGGTGTGAAGCAGAT	GACTTCCTTCACGATTTCATCGTAA	103
TKTKKF	ggatccACCAAGACGAAGAAGACGAAGA	-	-
TKTKKR	agatctCGTCTTCGTCTTGGTCTTCTTC	-	-
T-DNA LBR	CGAATTAATTCGGCGTTAATTCAGTAC	-	-
T-DNA RBF	GCAGCCTGAATGGCGAATGCTAGAG	-	-
35S::TKTKK1-14	GATTCTGTGTTGTTCTTTCCAAC	GATCAATCAAAGTCAACTGTTCTG	1152
35S::TKTKK1-21	GCTTCACGAGAAAAGAACGTGAC	GTAAAGACGCGAGGTTATACGTAC	1017
35S::TKTKK2-46	GAAAGGCAGAGAAAGGGAGAGAG	CAAATTATTTGATTTCCCAACAACTTG	936
35S::TKTKK3-5	GTACGTTAACTATTACTAAGAACCG	GGCTTGAAGTGTTTTCACTAAATC	988
